# Studies of xenobiotic-induced gut microbiota dysbiosis: from correlation to mechanisms

**DOI:** 10.1080/19490976.2021.1921912

**Published:** 2021-07-27

**Authors:** Liang Chi, Pengcheng Tu, Hongyu Ru, Kun Lu

**Affiliations:** Department of Environmental Sciences and Engineering, University of North Carolina at Chapel Hill, NC, United States

**Keywords:** Gut microbiota, xenobiotics, dysbiosis, host–microbiota interactions

## Abstract

Environmental chemicals can alter gut microbial community composition, known as dysbiosis. However, the gut microbiota is a highly dynamic system and its functions are still largely underexplored. Likewise, it is unclear whether xenobiotic exposure affects host health through impairing host–microbiota interactions. Answers to this question not only can lead to a more precise understanding of the toxic effects of xenobiotics but also can provide new targets for the development of new therapeutic strategies. Here, we aim to identify the major challenges in the field of microbiota-exposure research and highlight the need to exam the health effects of xenobiotic-induced gut microbiota dysbiosis in host bodies. Although the changes of gut microbiota frequently co-occur with the xenobiotic exposure, the causal relationship of xenobiotic-induced microbiota dysbiosis and diseases is rarely established. The high dynamics of the gut microbiota and the complex interactions among exposure, microbiota, and host, are the major challenges to decipher the specific health effects of microbiota dysbiosis. The next stage of study needs to combine various technologies to precisely assess the xenobiotic-induced gut microbiota perturbation and the subsequent health effects in host bodies. The exposure, gut microbiota dysbiosis, and disease outcomes have to be causally linked. Many microbiota–host interactions are established by previous studies, including signaling metabolites and response pathways in the host, which may use as start points for future research to examine the mechanistic interactions of exposure, gut microbiota, and host health. In conclusion, to precisely understand the toxicity of xenobiotics and develop microbiota-based therapies, the causal and mechanistic links of exposure and microbiota dysbiosis have to be established in the next stage study.

## Introduction

Xenobiotics, such as heavy metals, pesticides, antibiotics, and food additives, can cause adverse effects on human health. The mammalian gut microbiota plays a critical role in food fiber digestion, energy metabolism, immune system development, xenobiotic biotransformation and so on, and it has been characterized as an “exteriorized organ”.^1^^, 2^ Considering the important roles of the gut microbiota in host health, the effects of xenobiotics on the gut microbiota are extensively explored in the last two decades, and accumulating evidence indicates that many xenobiotics can profoundly perturb the gut microbiota composition to affect host health status.^3^ For example, artificial sweeteners can cause glucose intolerance by disturbing the gut microbiota in mice.^4^ However, the gut microbiota community is a highly dynamic system, and numerous factors, such as host genotype, diet, age, and host lifestyles, can significantly alter gut microbiota composition.^5–7^ The inter-individual and intra-individual variation of the gut microbiota is a pervasive phenomenon. Therefore, although exposure of multiple xenobiotics can shift gut microbiota, the changed gut microbiota is not necessary to cause adverse health effects in host bodies. Many studies indicate the association between xenobiotic-perturbed gut microbiota and host diseases, but the causality, for most of the compounds, is still unestablished. To better assess the effects of xenobiotic exposure on the gut microbiota as well as the subsequent health effects, we need to explore which specific functions of gut microbiota are impaired during exposure. The aims of this review are to summarize the current knowledge of the xenobiotic-driven gut microbiota dysbiosis, discuss the challenges and principles of future studies, and highlight the demonstrated mechanistic pathways of gut microbiota–host interactions to provide potential future research directions.

## Xenobiotic exposure and gut microbiota dysbiosis

### Antibiotics

Since the 20th century, antibiotics have been extensively produced and used to treat bacterial infections, which have saved millions of lives. However, many studies reveal that antibiotics increasingly accumulate in natural environments, including soil and aquatic environments.^8–10^ With the important roles of the gut microbiota in host health being continuously recognized, the profound and persistent impacts of antibiotic treatment on human gut microbiota have been given special attention.^11,12^ Various antibiotics can cause gut microbiota perturbation, including vancomycin, ampicillin, streptomycin, and metronidazole.^13–16^ Vancomycin, for example, can reduce the microbial diversity, decrease the gram-positive bacteria, and cause the compensatory increase of gram-negative bacteria.^13,17^ The antibiotic-shifted gut microbiota is associated with multiple diseases. A typical example is that antibiotic treatment affects *Clostridium difficile* colonization and host susceptibility to *C. difficile* infection.^18,19^ Clindamycin-induced gut microbiota dysbiosis is associated with long-lasting susceptibility to *Clostridium difficile* infection.^20^ In addition, vancomycin-induced gut microbiota dysbiosis is associated with decreased insulin sensitivity by modifying bile acid metabolism.^13^ Another study has found a cocktail of antibiotics perturbed the gut microbiota to disrupt gut redox dynamics.^21^ On the other hand, antibiotics also could reduce disease risk by modifying gut microbiota. For example, rifaximin treatment can improve the level of beneficial bacteria, such as Bifidobacteria and Lactobacilli, to reduce disease risk.^22^ Depletion of Firmicutes and Bacteroidetes caused by vancomycin and bacitracin ameliorates insulin resistance in mice with diet-induced obesity.^23^

### Heavy metals

Heavy metals are environmental pollutants that affect the health of millions of people in the world.^24^ Interactions between gut microbiota and heavy metals have been studied since the last century, mainly focusing on the gut bacteria-performed heavy metal biotransformation.^25–27^ Recently, the heavy metal-driven gut microbiota dysbiosis is also investigated. For example, inorganic arsenic exposure has been demonstrated to change gut microbiota community structure, functional gene patterns as well as the metabolome profiles.^28–30^ Our previous study has demonstrated that chronic arsenic exposure changed community diversity, reduced the relative abundance of Firmicutes, and shifted the carbohydrate metabolic gene patterns in female mice.^29^ Moreover, the sex-dependent effects of arsenic exposure on the gut microbiota are observed.^31,32^ The effects of lead exposure are also explored, and both acute and chronic lead exposure can cause gut microbiota dysbiosis in animal studies.^33–37^ Short-term lead exposure significantly alters the richness and diversity of the gut microbiota, increases the abundance of α-Proteobacteria, and decreases the abundance of Firmicutes in zebrafish.^33^ But another study used mouse model reveals that chronic lead exposure-perturbed gut microbiota is characterized by decreased Bacteroidetes and increased Firmicutes.^34^ Chronic lead exposure can shift the gut microbiota metabolic profiles to change the abundance of functional metabolites, such as amino acids, bile acids, and tricarboxylic acid (TCA) cycle-associated metabolites.^34,35^ Perinatal lead exposure can induce gut microbiota dysbiosis by increasing Firmicutes and decreasing Bacteroidetes.^36^ Likewise, cadmium exposure can also perturb normal gut microbiota community compositions.^38,39^ Short-term cadmium exposure can inhibit the growth of Bacteroidetes as well as some probiotics, such as *Lactobacillus* and *Bifidobacterium*, and reduce the copy number of short-chain fatty acid (SCFA)-associated genes.^38^ Another study demonstrates the abundance of Firmicutes is decreased in cadmium-treated mice.^39^ Gut microbiota dysbiosis caused by early-life cadmium exposure is associated with the exposure-induced fat accumulation in male mice.^40^

### Pesticides

Pesticide contamination is another serious threat to public health, and the effects of pesticide exposure on the gut microbiota are explored recently. For example, a recent study on honey bees has revealed that glyphosate, one of the most popular herbicides, specifically inhibited the activity of 5-enolpyruvylshikimate-3-phosphate synthase enzyme in the shikimate pathway of gut microbiota.^41^ This inhibition causes the decline of beneficial gut microbiota with this enzyme and increases the mortality of bees infected with pathogen *Serratia marcescens*. Glyphosate-induced gut microbiota dysbiosis has also been associated with neurobehavioral alterations.^42,43^ Organophosphate insecticides are another group of extensively used pesticides, and their effects on the gut microbiota are also investigated. For example, diazinon is found to cause sex-specific effects on mouse gut microbiota, differentially changing the bacterial components, functional gene composition as well as fecal metabolite profiles in male and female mice.^44^ Trichlorfon exposure can decrease the abundance of *Lactobacillus* in Japanese quail.^45^ Moreover, a recent study revealed that organophosphate-induced hyperglycemia was directly associated with the altered gut microbiome.^46^ Organophosphate exposure enriches the xenobiotic biodegrading genes in gut microbiome which promote the gut microbiota to utilize organophosphate to produce acetic acid. The increased acetic acid then enhances the gluconeogenesis in host bodies and finally results in glucose intolerance. Many other pesticides are also found to induce gut microbiota dysbiosis, such as 2,4-D,^47^ clorpyrifos,^48,49^ imazalil^50–52^ and so on. Recent studies of pesticide effects on the gut microbiota is well summarized by a recent review.^53^

### Artificial sweeteners

Non-caloric artificial sweeteners are widely used in the food industry to enhance sweet taste without the associated high energy content of caloric sugars. Artificial sweeteners generally have low absorption and metabolism rates, and in a long time, they are considered to be harmless to humans.^54^ However, some studies indicate that artificial sweeteners can perturb gut microbiota and cause adverse effects on host health. For example, chronic saccharin consumption can exacerbate glucose intolerance in mice by mediating gut microbiota.^4^ Saccharin consumption increases Bacteroidetes phylum, reduced Firmicutes phylum, and also decreases SCFA production. Our previous study has found saccharin-induced gut microbiota shift was associated with liver inflammation.^55^ SUCRAM, consisting of saccharin and neohesperidin dihydrochalcone, has been demonstrated to induce the growth of *Lactobacillus*.^56,57^ Likewise, sucralose, the most popular artificial sweetener, also can alter gut microbiota. Chronic sucralose consumption causes a shift of a series of gut microbiota genus and enriches the pro-inflammatory genes in mouse gut microbiome.^58^ A recent study found that sucralose caused gut microbiota dysbiosis was associated with a high level of hepatic cholesterol and altered bile acid profiles.^59^ Some animal studies find that aspartame and acesulfame-K could perturb normal gut microbiota composition,^60,61^ and an epidemiologic study also reveals that human gut microbiota diversity is different between aspartame or acesulfame-K consumers and non-consumers.^62^ A summary of the effects of artificial sweeteners on the gut microbiota can be found in two recent reviews.^63,64^

### Others

The number of compounds that can perturb the gut microbiota is much more than those we listed above. For example, numerous of non-antibiotic drugs have extensive effects on the gut microbiota.^65,66^ The interaction of gut microbiota and drugs is tightly associated with the drug side-effects in host bodies. In addition, some environmental pollutants, such as polychlorinated biphenyls (PCBs),^67,68^ polycyclic aromatic hydrocarbons (PAHs),^69^ and triclosan,^70–72^ can disturb the gut microbiota community and affect host health. Our previous studies also demonstrated that nicotine consumption shifted the gut microbiota composition in mice.^73^ It can be anticipated that compounds that impact gut microbiota will be continuously reported.

## Current difficulties and limitations in xenobiotics–microbiota studies

**Figure 1.**
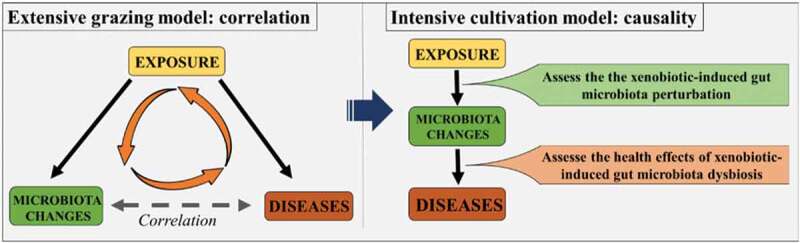
Scheme to show the differences between “extensive grazing model” and “intensive cultivation model”

Previous studies have evaluated the gut microbiota perturbation induced by a series of xenobiotics, but in most cases, we only demonstrated the correlation of xenobiotic exposure, gut microbiota dysbiosis, and disease outcomes, that unexposed and exposed subjects had different gut microbiota profiles. These studies can be regarded as “extensive grazing model” which show the potential effects of those xenobiotics on gut microbiota and provides basic information as well as the theoretical basis for the subsequent research. But to further understand how xenobiotic-induced gut microbiota dysbiosis affects our health and to develop new microbiota-based intervention approaches, “extensive grazing model” has transfer to “intensive cultivation model” in which the causality between xenobiotic-perturbed gut microbiota and host diseases are determined (Figure 1). However, there are many challenges to establish the causal relationship of xenobiotic exposure, gut microbiota dysbiosis, and host diseases.

First, the gut microbiota community is a highly dynamic and complex system, so the specific roles of each type of gut microorganisms in host homeostasis are unclear. Moreover, many recent studies reveal that the same species from different humans have different characters and can cause very different health impacts on host bodies.^74,75^ Therefore, the exact definition of “healthy/normal gut microbiota” is still undetermined in many aspects, though many studies have conducted to try to define it.^76,77^ In addition, notably, although gut microbiota is considered as an extra organ, gut microbiota damage does not necessarily affect our health; after all, they are not a true part of our bodies, which means that xenobiotic-shifted gut microbiota is not always detrimental to host health. It is largely unknown that when gut microbiota shift is bad and can cause adverse health effects on host bodies.

Secondly, functional redundancy is ubiquitous in the gut microbiota community. For example, butyrate-producing bacteria exist both in the phylum Bacteroidetes and Firmicutes.^78,79^ Therefore, community composition changes are not always consistent with functional dysbiosis. But, without knowing the functional perturbation of the gut microbiota, it is hard to evaluate the associated health effects in host bodies. Thus, unless we know the specific function of each bacteria, only investigating the gut microbiota composition changed by xenobiotics wouldn’t improve our understanding of the associated health effects.

Thirdly, xenobiotic effects on host bodies and gut microbiota may occur simultaneously. Therefore, although many studies detect gut microbiota shift and host health deterioration during xenobiotic exposure, we actually cannot determine whether the xenobiotic-shifted gut microbiota impairs host health, or the xenobiotic-deteriorated host responses affect the gut microbiota, or this is a dynamic and bi-directional process. Currently, cause and effect have not been demonstrated for most of the studies.

Last but not least, many factors could cause the detectable fluctuation of community composition, including but not limited to diet, bedding caging, sampling time (gut microbiota rhythm), DNA extracting method, and sequencing data analysis. Craigl L. Franklin and other researchers have done some valuable work on the impact of some “peripheral factors” on gut microbiota composition.^80–82^ But, in general, the effects of those factors on experiments are rarely quantified and controlled between different studies or even in a single study, which challenges the reproducibility of microbiota-associated studies. Standardization of experimental design remains a critical need in the field.

In summary, to determine the health effects of xenobiotic-induced gut microbiota perturbation, we have to distinguish the natural fluctuation and dysbiosis of the gut microbiota. The former one would not affect host health status, but the gut microbiota dysbiosis has to be linked with adverse health effects in host bodies. To build the links, we need a more refined view to investigate the effects of xenobiotic exposure on the gut microbiota. In other words, we need to explore which specific aspects of gut microbiota are changed by xenobiotics and whether and how these changes affect host health.

## Important research principles, strategies, and technologies

### Precisely assessing the xenobiotic-induced gut microbiota perturbation

Precisely assessing the xenobiotic-induced gut microbiota perturbation is the prerequisite and foundation of linking gut microbiota dysbiosis with host health conditions and developing microbiota-directed therapies (Figure 1). However, as the high dynamics of the gut microbiota, many factors can influence the gut microbiota composition. Although controlling the interferences of unrelated variables are required in most of the experiments, for gut microbiota-related studies, it needs to be especially emphasized and concerned, that some “peripheral factors” need to be strictly controlled or normalized, such as diet, drinking water, host genetics, and so on.^80–82^ Notably, the cage effects should be highly concerned, because the mice in the same cage frequently share their gut microbiota and tend to have similar gut microbiota profiles.^83^ Cage effects decrease the efficiency of individual replicates, and thus multiple cages need to be set in the same experimental group to avoid the false positive or negative results caused by the cage-effects.

In addition, gut microbiota variation in different individuals or groups cannot be completely avoided, which requires us to identify the xenobiotic-caused gut microbiota changes from the neutral fluctuation. How to efficiently filter the true signals from noise is a critical question that needs to be answered. 16S rRNA sequencing, metagenomics, metatranscriptomics, metaproteomics, and metabolomics are powerful technologies that can efficiently identify the changes of gut microbiota,^84,85^ but also can induce many false-positive results. Combining multiple technologies can provide a better understanding of the xenobiotic-induced gut microbiota dysbiosis. The *in-vitro* devices, such as batch-culturing systems, simulators of the human intestinal microbial ecosystem (SHIME), the chemostat-type simulators, and gut-on-chip devices can also help to evaluate the effects of xenobiotics on gut microbiota,^86^ although it is still a challenge to exactly mimic the true physiological conditions to maintain the gut microbiota community. In addition, the advance of system biology and computational biology may also provide some other solutions to identify the exposure-induced gut microbiota dysbiosis. For example, recent studies in Dr. Gordon’s group successfully utilized the Random Forests, a machine learning approach, to identify the age-discriminatory taxa in children, which allowed to quantitatively assess the unhealthy level of the gut microbiota.^87,88^ Another recent study defined the “ecogroup” which used a series of conserved covarying taxa to evaluate the alteration of the gut microbiota under different scenarios.^89^ Comparing using the changes of discrete community components, the variation of ecogroup may better reflect the functional changes of the gut microbiota between different groups.

### Assessing the health effects of xenobiotic-induced gut microbiota dysbiosis

Comparing with assessing the effects of xenobiotics on the gut microbiota, assessing the specific health effects of xenobiotic-induced gut microbiota dysbiosis are more concerned by toxicologists. Many studies detected the differential gut bacterial profiles in the untreated group and xenobiotic-treated group. However, both the xenobiotics exposure and the exposure-caused gut microbiota dysbiosis can affect host health, and host conditions also can affect gut microbiota. Studies based on traditional exposure study design cannot well determine the causal link of xenobiotic exposure, gut microbiota dysbiosis, and host health impairments.

Germ-free animals and antibiotic-treated animals are powerful tools to explore whether the gut microbiota plays a role in xenobiotic-associated adverse health effects on host bodies. They are analogous to the “gut microbiota knock-out” and “gut microbiota knock-down” animals. By comparing the different responses of conventional-raised animals and germ-free/antibiotic-treated animals, we can determine whether the presence of gut microbiota can influence the toxic effects of xenobiotics. In addition, gut microbiota transplantation is a key step. By transplanting the xenobiotic-exposed gut microbiota to healthy germ-free or antibiotic-treated animals, we can determine the specific health effects of exposure-driven gut microbiota dysbiosis in host bodies. However, it should be noticed that germ-free animals have largely different physiological conditions with conventional-raised animals, especially of their immune system, and antibiotic treatment may also affect host body conditions. Moreover, transplanting efficiency needs to be carefully evaluated.

Transplanting the exposed gut microbiota to germ-free animals is helpful to evaluate the overall health effects of the whole community, but it still cannot tell us which components are the key factors. After all, not all of xenobiotic-induced changes in gut microbiota will affect host health. Decipher the “black box”, that to find the key functional components and pinpoint the key perturbation events, is the critical step to promote the development of microbiota-directed new therapies to treat exposure-caused diseases. However, since our current knowledge about the function of each bacterial components in the gut microbiota is still largely limited, it is still a big challenge to identify the functional taxa, genes, and metabolites. An executable experimental process is first to select those taxa, genes, or metabolites that dramatically changed by exposure as candidates and then validate them by complementary experiments.^90^

## Metabolite-driven microbiota–host interactions

**Figure 2.**
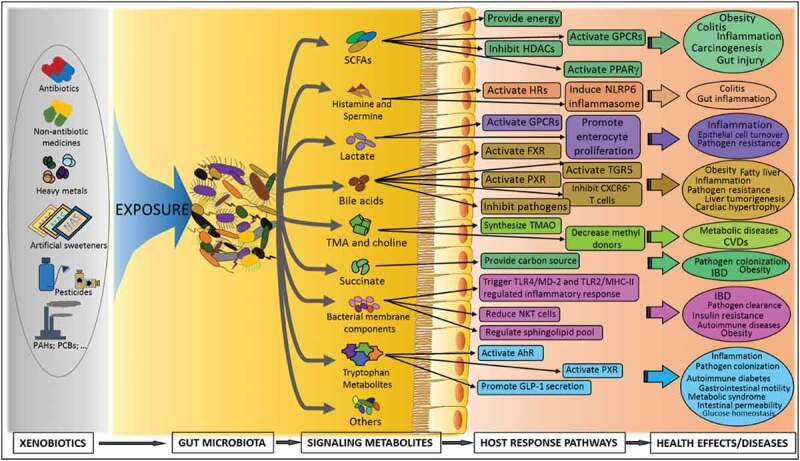
Schematic representation of potential mechanistic links of xenobiotic-induced gut microbiota dysbiosis and adverse health effects. PAHs: polycyclic aromatic hydrocarbons; PCBs: polychlorinated biphenyls; SCFAs: short-chain fatty acids; TMA: trimethylamine; TMAO: trimethylamine-N-oxide; GPCRs: G protein-coupled receptors; HDACs: histone deacetylases; PPARγ: peroxisome proliferator-activated receptor gamma; HR: histamine receptor; IBD: inflammatory bowel disease; TGR5: the G protein-coupled bile acid receptor 1; CVDs: cardiovascular diseases

Microbiota-derived metabolites play a central role in microbiota–host interactions,^2,91^ and previous studies have found various of key metabolites functioning as signaling molecules by which the microbiota affects host homeostasis. Although the whole picture of the microbiota–host interactions is not fully understood, many mechanistic pathways about how microbiota-derived metabolites affect host health have been found by previous studies. Those mechanistic pathways causally link the gut microbiota dysbiosis and host disease outcomes. In the following, we summarize well-demonstrated metabolite-driven microbiota–host interactions, which could serve as the starting points and measurable indexes to examine the xenobiotic-induced gut microbiota dysbiosis and the associated health effects (Figure 2).

### SCFAs

SCFAs, mainly including acetate, butyrate, and propionate, are the end products of anaerobic dietary fiber fermentation. As one group of the most abundant microbiota-synthesized metabolites, SCFAs have been found to play important roles in various physiological processes, especially in energy supplement, immune regulation, gut barrier integrity maintenance, pathogen resistance, and gut-brain axis.

#### SCFAs function as energy sources to influence host energy homeostasis

Most of SCFAs are absorbed in the intestinal epithelium in ceca and colons. SCFAs, especially butyrate, are the main energy source of colonocytes and provide around 10% of daily caloric in humans estimated by a previous study.^92^ The rest SCFAs are mainly metabolized in livers as energy source or used to synthesize host endogenous metabolites. For example, propionate can be utilized in gluconeogenesis to synthesize glucose in livers.^93,94^ SCFAs regulated energy homeostasis has been correlated with obesity, and previous studies found that mouse and human subjects with obesity have higher levels of intestinal SCFAs than in lean groups.^95,96^

#### SCFAs function as G-protein-coupled receptor (GPCRs) ligands to regulate immune response

Activating GPCRs is a critical mechanism of SCFA-conducted immunity regulation.^97,98^ GPR43 is a receptor that can be activated by acetate, butyrate, propionate. SCFAs activated GPR43 signaling promotes the proliferation of CD4^+^ regulatory T (T_reg_) cells with enhanced Foxp3 and IL-10 levels, which improves colonic homeostasis and protects against colitis.^99^ Acetate-activated GPR43 can protect non-obese diabetic mice by increasing the frequency of T_reg_ cells but reducing autoreactive T cells in peripheral tissues.^100^ Another study found that SCFA-activated GPR43 promoted the NLRP3 inflammasome activation by stimulating K^+^ efflux and hyperpolarization, which provides health benefits to against colitis.^101^ Moreover, previous studies found that SCFA-mediated GPR43 signaling suppressed insulin signaling in adipocytes and also increased the level of TNF-α in anti-inflammatory M2-type macrophages in adipose tissues, that both of them can inhibit fat accumulation.^102,103^ GPR41 is another receptor of SCFAs. Propionate-activated GPR41 regulates the generation of macrophages and dendritic cell precursors to protect against allergic inflammation in the lung.^104^ In human renal cortical epithelial cells, SCFA-activated GPR41 and GPR43 can reduce TNF-α-induced MCP-1 expression by suppressing p38 and JNK phosphorylation.^105^ In addition, butyrate can specifically activate GPR109A. In colonic macrophages and dendritic cells, butyrate-activated GPR109A promotes the differentiation of T_reg_ cells and IL-10-producing T cells, which inhibits colonic inflammation and carcinogenesis.^106^ The regulatory role of butyrate on T_reg_ cells by GPR109A can also ameliorate gastrointestinal injury during graft versus host disease.^107^ A previous study demonstrated that SCFA-mediated GPR109A and GPR43 activation played a role in food antigens tolerance by increasing mucosal CD103^+^ dendritic cells.^108^

#### SCFAs regulate host homeostasis through inhibiting histone deacetylases (HDACs)

Butyrate and propionate can inhibit the HDAC to promote the Foxp3 protein acetylation and then increase the generation of T_reg_ cells.^109^ Likewise, butyrate-induced HDAC inhibition can reduce the LPS-stimulated pro-inflammatory mediators in intestinal macrophages, which may protect against ulcerative colitis or Crohn’s disease.^110^ Another study found that butyrate-regulated HDAC3 inhibition in macrophages reduced the mTOR kinase activity but increased the antimicrobial peptide production, which enhanced the host antimicrobial capability.^111^ In the context of graft-versus-host disease, butyrate-promoted HDAC3 inhibition benefits the gut barrier integrity.^112^

#### Other interactional mechanisms

In addition to the three main mechanistic pathways, SCFAs also can affect host health by some other approaches. For example, butyrate can activate the peroxisome proliferator-activated receptor γ (PPAR-γ) in colonic epithelial cells to promote the β-oxidation, which limits the aerobic pathogen expansion, such as *Escherichia* and *Salmonella*.^113,114^ On the other hand, however, SCFAs can down-regulate the PPAR-γ expression in liver and adipose tissue to inhibit lipid synthesis but increase lipid utilization, which helps to prevent high-fat-diet-induced obesity, reduce hepatic steatosis, and improve insulin sensitivity.^115^ A previous study found that propionate could reduce the stability of HilD by disturbing its post-translationally modification and thus repress the *Salmonella* invasion.^116^

### Bile acids

Bile acids are originally synthesized from cholesterol in livers known as primary bile acids, stored in gall bladders and then secreted to small intestines to solubilize lipid and fat-soluble vitamins as potent detergents after a meal. Most of the bile acids can be reabsorbed and backed to our bodies, which is called bile acid enterohepatic circulation. Gut bacteria can hydrolyze the amino acid residues (taurine or glycine) in conjugated bile acids to generate free bile acids. Moreover, some gut bacteria also can synthesize secondary bile acids utilizing primary bile acids.^117^ Gut bacteria-performed bile acid biotransformation is a critical interaction between the gut microbiota and host, which is not only required to maintain the bile acid homeostasis but also provides key metabolic signaling to multiple tissues in host bodies.^118^

**The gut microbiota influences host homeostasis by affecting bile acid-regulated farnesoid X receptor (FXR) signaling**

FXR is a transcription factor which presents in multiple tissues and regulates the expression of a wide range of target genes.^119^ The most potent endogenous ligands for FXR include chenodeoxycholic acid (CDCA), lithocholic acid (LCA), deoxycholic acid (DCA), and cholic acid (CA), that LCA and DCA are the two secondary bile acids synthesized by gut microbiota.^120^ In addition, tauro-β-muricholic acid (TβMCA) has been identified as an antagonist of FXR. Previous studies demonstrated the regulatory roles of gut microbiota on FXR signaling. Germ-free mice display a higher level of TβMCA and a lower activation level of FXR signaling than conventional mice.^121^ Bile salt hydrolase (BSH) activity in gut microbiota, which catalyzes the de-conjugation of conjugated bile acids, is correlated with the TβMCA level as well as the FXR activation.^122^ The FXR signal in livers and ilea inhibits bile acid synthesis.^123^ Gut microbiota-mediated bile acid metabolism and FXR activation play an important role in the regulation of bile acid pool size.^118^ In addition, intestinal FXR signaling is required for gut microbiota-associated lipid accumulation and obesity. Inhibiting intestinal FXR signaling by treating with antibiotics or tempol can reduce high-fat diet-induced hepatic triglyceride accumulation.^124^ Modulating gut bacteria to decrease intestinal FXR activation can ameliorate the high-fat diet-induced obesity.^122,125^ Moreover, DCA-activated intestinal FXR signaling inhibits prostaglandin E2 production and promotes crypt regeneration, which benefits the colonic wound repair.^126^

**The gut microbiota influences host homeostasis by affecting bile acid-regulated TGR5 signaling activation**

TGR5 is another transcription factor expressing in a wide range of tissues, which is mainly activated by LCA, DCA, and tauroursodeoxycholic acid (TUDCA), the secondary bile acids synthesized by gut microbiota.^127,128^ TUDCA has shown anti-inflammatory effects by activating TGR5 in the nervous system.^128,129^ An *in vitro* study reveals that LCA-activated TGR5 can ameliorate cardiac hypertrophy.^130^

#### Other interactional pathways

There are some other mechanisms of the gut microbiota-regulated bile acid metabolism affecting host health status. The gut microbiota-conducted taurine deconjugation can activate the NOD-like receptor family pyrin domain containing 6 (NLRP6) inflammasome and increase IL-18 level to promote intestinal inflammation ^132^. Secondary bile acids, such as LCA and DCA, are known by their high cytotoxicity and carcinogenic effects. DCA has been demonstrated to inhibit tumor-suppressing CXCR6+ natural killer T cells and promote liver tumorigenesis.^131,132^ Hepatic Pregnane X receptor (PXR) can be activated by LCA to prevent LCA-caused liver damage.^133^ On the other hand, however, the high toxicity of secondary bile acids also exhibits beneficial effects on host by preventing the colonization of certain pathogens, such as *Clostridium difficile*.^134^

### Tryptophan metabolites

Tryptophan is an essential aromatic amino acid which is required for protein synthesis and some key metabolite biosynthesis in mammals. In the last decade, gut microbiota-derived tryptophan metabolism has been extensively studied and it reveals that tryptophan and associated metabolic products play an important role in microbiota–host interactions.

#### Indole derivatives from tryptophan activate the aryl hydrocarbon receptor (AHR)

Gut microbiota can metabolize tryptophan to multiple indole-containing metabolites, such as indole-3-acetic acid (IAA), indole-3-lactic acid (ILA), and indole-3-propionic acid (IPA), which are important AHR agonists.^135,136^ For example, *Lactobacillus reuteri* produced ILA can down-regulate transcription factor Thpok to promote the differentiation of CD4^+^ T cells into CD4^+^CD8αα^+^ double-positive intraepithelial lymphocytes by activating AHR, which benefits to intestinal inflammation.^137^ Indole-3-aldehyde (IAld) from *Lactobacillus reuteri* can activate AHR, which promotes IL-22 production to inhibit mucosal inflammation and resistant fungus *Candida albicans* colonization.^138^ IAld-induced AHR activation also promotes IL-22 secretion in pancreatic innate lymphoid cells to protect against autoimmune diabetes.^139^ Likewise, IAA-induced AHR activation can attenuate inflammatory responses in macrophages and hepatocytes.^138^ Moreover, bacteria-derived indoxyl-3-sulfate, IPA and IAld also can limit central nervous system (CNS) inflammation by activating AHR in astrocytes.^140^ A previous study revealed that decreased indole derivatives from tryptophan caused a low AHR activation level which was associated with metabolic syndrome, and rescuing AHR activation could significantly improve metabolic dysbiosis.^141^

#### Other mechanic pathways that indole derivatives performed microbiota–host interactions

In addition to AHR activation, tryptophan-sourced indole derivatives also can modulate host homeostasis by other pathways. For example, IPA can activate PXR to promote the gene expression of tight junctional protein and downregulate enterocyte TNF-α, which decreases intestinal permeability and inflammation.^142^ Another study finds that acute treatment with indole promotes the secretion of glucagon-like peptide-1 (GLP-1) in colonic L cells by modulating the voltage-gated K^+^ channel- and Ca^+^-dependent action potentials, but continuous exposure to indole reduced GLP-1 secretion by blocking NADH dehydrogenase to decrease ATP synthesis.^143^

#### Tryptophan-derived neurotransmitters

Gut microbiota also can metabolize tryptophan to different neurotransmitters, such as tryptamine and serotonin.^144,145^ Tryptamine is a product of tryptophan catabolism functioning as a β-arylamine neurotransmitter. *Clostridium sporogenes*-produced tryptamine by decarboxylating tryptophan induces ion secretion in intestinal epithelial cells which could affect gastrointestinal motility.^145^ Likewise, another study demonstrated that tryptamine could activate GPCR serotonin receptor-4 to promote fluid secretion and accelerate gut transit.^146^ Serotonin is another key neurotransmitter in the gut-brain-axis.^144^ A previous study demonstrated that indigenous spore-forming bacteria in gut microbiota promoted serotonin biosynthesis in colonic enterochromaffin cells, which increased the gastrointestinal motility and enhanced platelet activation and aggregation.^147^ The gut microbiota-regulated peripheral serotonin synthesis plays a mediatory role in host glucose homeostasis.^148^ However, how microbiota-derived serotonin affecting neuron system is still largely unclear.

### Membrane components of gut microbiota

Multiple gut microbial membrane components can deeply influence host metabolism, especially regulating host immune response. Lipopolysaccharides (LPS), a cell wall component of gram-negative bacteria, is possibly the most investigated potent activator of innate immune signaling, and LPS plays an important role in gut microbiota-derived inflammatory responses. The TLR4/MD-2 complex at the cell surface and endosomes is the receptor of LPS, which can be activated by LPS and trigger the downstream immune responses, such as inducing MAP kinases and NF-κB and activating p38 and JNK.^149,150^ LPS-induced host inflammation is associated with various diseases, such as IBD inflammatory bowel diseases (IBD), obesity, insulin resistance, and autoimmune and allergic diseases.^151–153^

In addition, capsular polysaccharide A is deeply involved in the *Bacteroides fragilis*-modulated immune responses. Polysaccharide A can bind with MHC-II and the TLR2 receptor in plasmacytoid dendritic cells and then stimulate CD4^+^ T_reg_ cells to secrete anti-inflammatory cytokine IL-10, which helps to protect against colitis.^154^ A previous study found that polysaccharide A stimulated the suppressive CD4(+)CD45RB(low) effector/memory T cells by forming polysaccharide A-MHCII complex, which induced anti-inflammatory responses.^155^ Sphingolipids are another group of bacterial membrane components that play a role in the functional interaction between gut microbiota and host metabolism. For example, membrane glycosphingolipids from some *Sphingomonas spp*. can activate natural killer T (NKT) cells and promote the cytokine release which is a benefit to the pathogen clearance during infection.^156,157^ In contrast, α-galactosylceramide from *B. fragilis* can reduce colonic invariant NKT (iNKT) cells which attenuates pro-inflammatory responses and protect against colitis.^158^ A recent study reveals that *Bacteroides*-derived sphingolipids regulate the pool of host sphingolipids and is correlated with IBD.^159^

### Histamine

Histamine can be synthesized by host cells as well as various histamine-secreting bacteria and four different receptors can be activated by histamine, including H1R, H2R, H3R, and H4R.^160^ Histamine produced by the gut microbiota has immunomodulatory activity. For example, in human monocytoid cells, *Lactobacillus reuteri*-produced histamine can activate

histamine H2 receptor (H2R) to elevate cAMP levels and inhibit the downstream MEK/ERK MAPK signaling, which inhibits the TLR-induced TNF-α production.^161^ Histamine-induced H2R activation plays a critical role in *Lactobacillus reuteri*-driven suppression of intestinal inflammation.^162^ Another study found microbiota-associated histamine as well as spermine reduced NLRP6 inflammasome assembly and decreased IL-18 secretion to regulate the host immune homeostasis and affect the host susceptibility to colitis.^163^

### Lactate

Lactate is a ubiquitous metabolite in the gut that can be synthesized by some gut bacteria, especially by *Lactobacillus* species. Microbiota-derived lactate accelerates colon epithelial cell turnover in starvation-refed mice by promoting the enterocyte hyperproliferation.^101^ Lactate also has immunomodulatory activity that can inhibit NF-κB activation^164^ and regulate the TLR signal in intestines.^165^ Moreover, lactate can specifically activate GPR81,^166^ which can reduce TLR4-dependent inflammation.^167^ A recent study found lactate produced by *Bifidobacterium* and *Lactobacillus spp*. promoted the intestinal stem-cell proliferation in a GPR81-dependent manner.^168^ In addition, another study indicated that bacteria-derived lactate and pyruvate induced dendrite protrusion in CX3CR1^+^ cells by activating GPR31, which enhanced the host resistance to intestinal *Salmonella* infection.^169^ Lactate can be utilized to synthesize butyrate in gut microbiota, which may also affect host homeostasis.^170^

### Trimethylamine (TMA) and choline

Choline, phosphatidylcholine, and L-carnitine can be metabolized to trimethylamine (TMA) by choline TMA-lyase in some gut microbes, which can be further oxidized to trimethylamine-N-oxide (TMAO) by host hepatic flavin monooxygenase.^171^ TMAO is known as its proatherogenic effect that enhances atherosclerosis and increases the risk of cardiovascular diseases.^171,172^ Microbiota-derived TMAO can enhance stimulus-dependent platelet activation by promoting Ca^+^ release which potentiates thrombosis potential.^173^ High serum levels of TMAO are also positively associated with impaired renal function, liver steatosis and diabetes.^174–176^ In addition, bacteria-conducted choline metabolism can modulate host homeostasis by affecting host epigenetic programming, that choline consumption by the gut microbiota decreases the availability of methyl-donor metabolites in host bodies resulting in the alteration of global DNA methylation patterns to increase anxiety as well as the susceptibility to metabolic disease.^177^

### Succinate

Succinate is a common metabolite participating in the TCA cycle and it can also function as a signaling molecule to mediate microbiota–host interactions, especially by affecting pathogen infection. For example, the *Salmonella enterica* serovar Typhimurium, another enteric bacterial pathogen, can get a competitive growth advantage by utilizing succinate as carbon source.^178^ Disturbing normal gut microbiota community by antibiotic and polyethylene glycol treatment increases the succinate level in intestines, which can be utilized as an energy source by *Clostridium difficile* and finally promote the pathogen colonization.^19^ In addition, *Bacteroides thetaiotaomicron*-produced succinate can promote the virulence gene expression in another pathogen Enterohemorrhagic *Escherichia coli* by activating the transcriptional regulator *Cra*.^179^ Succinate accumulation has also been associated with IBD and obesity.^180–183^

### Others

Extracellular ATP in the small intestinal lumen is another metabolic signal performing the interactions between gut microbiota and host. Microbiota-derived ATP is sensed by the ATP-gated ionotropic P2X7 receptor to limit the secretory IgA response in the small intestine by reducing Tfh cell activity, which is important to maintain the mucosal ecosystem homeostasis.^184^ Taurine can enhance the NLRP6 inflammasome-induced IL-18 secretion, which influences the host immune homeostasis and affected the host susceptibility to colitis.^163^ Ascorbate, a microbiota-derived metabolite associated with Crohn’s disease, can selectively inhibit activated human CD4^+^ effector T cells by suppressing their energy metabolism and inducing apoptosis.^185^

## Conclusions

In conclusion, the effects of xenobiotics on the gut microbiota are extensive and profound. However, our understanding of how those effects contribute to xenobiotic-induced toxic effects in the host is still very limited. The key needs are to precisely assess the xenobiotic-induced functional changes in gut microbiota and decipher which specific functional interactions/pathways between microbiota and host are altered by xenobiotics, which allow the following health effect evaluation. Currently, many mechanistic pathways of gut microbiota–host interactions have been found by previous studies, and more are expected to be discovered in the coming years. Linking those mechanistic pathways with xenobiotic exposure not only can elucidate the specific effects of xenobiotics on gut microbiota and help us better evaluate the toxicity and health effects of xenobiotics, but also can provide valuable targets for the development of microbiota-based new intervention approaches and therapies.

## References

[1] Baquero F, Nombela C. The microbiome as a human organ. Clin Microbiol Infect. 2012;18:2–19. doi:10.1111/j.1469-0691.2012.03916.x. 22647038

[2] Nicholson JK, Holmes E, Kinross J, Burcelin R, Gibson G, Jia W,Pettersson, S. Host-gut microbiota metabolic interactions. Science. 2012;336:1262–1267. 22674330 10.1126/science.1223813

[3] Maurice CF, Haiser HJ, Turnbaugh PJ. Xenobiotics shape the physiology and gene expression of the active human gut microbiome. Cell. 2013;152:39–50. 23332745 10.1016/j.cell.2012.10.052 PMC3552296

[4] Suez J, Korem T, Zeevi D, Zilberman-Schapira G, Thaiss CA, Maza O, Israeli D, Zmora N, Gilad S, Weinberger A, et al. Artificial sweeteners induce glucose intolerance by altering the gut microbiota. Nature. 2014;514:181–186. 25231862 10.1038/nature13793

[5] Conlon M, Bird A. The impact of diet and lifestyle on gut microbiota and human health. Nutrients. 2015;7:17–44. 10.3390/nu7010017 PMC4303825 25545101

[6] Kovacs A, Ben-Jacob N, Tayem H, Halperin E, Iraqi FA, Gophna U. Genotype is a stronger determinant than sex of the mouse gut microbiota. Microb Ecol. 2011;61:423–428. 21181142 10.1007/s00248-010-9787-2

[7] Yatsunenko T, Rey FE, Manary MJ, Trehan I, Dominguez-Bello MG, Contreras M, Magris M, Hidalgo G, Baldassano RN, Anokhin AP, et al. Human gut microbiome viewed across age and geography. Nature. 2012;486:222–227. 22699611 10.1038/nature11053 PMC3376388

[8] Kümmerer K. Significance of antibiotics in the environment. J Antimicrob Chemother. 2003;52:5–7. 12805262 10.1093/jac/dkg293

[9] Kümmerer K. Antibiotics in the aquatic environment–a review–part I. Chemosphere. 2009;75:417–434. 19185900 10.1016/j.chemosphere.2008.11.086

[10] Jechalke S, Heuer H, Siemens J, Amelung W, Smalla K. Fate and effects of veterinary antibiotics in soil. Trends Microbiol. 2014;22(9):536–545. doi:10.1016/j.tim.2014.05.005. 24950802

[11] Modi SR, Collins JJ, Relman DA. Antibiotics and the gut microbiota. The Journal of Clinical Investigation. 2014;124:4212–4218. 25271726 10.1172/JCI72333 PMC4191029

[12] Jernberg C, Löfmark S, Edlund C, Jansson JK. Long-term impacts of antibiotic exposure on the human intestinal microbiota. Microbiology. 2010;156:3216–3223. 20705661 10.1099/mic.0.040618-0

[13] Vrieze A, Out C, Fuentes S, Jonker L, Reuling I, Kootte RS, van Nood E, Holleman F, Knaapen M, Romijn JA, et al. Impact of oral vancomycin on gut microbiota, bile acid metabolism, and insulin sensitivity. J Hepatol. 2014;60:824–831. 24316517 10.1016/j.jhep.2013.11.034

[14] Membrez M, Blancher F, Jaquet M, Bibiloni R, Cani PD, Burcelin RG,Corthesy I, Macé K, Chou CJ. Gut microbiota modulation with norfloxacin and ampicillin enhances glucose tolerance in mice. Faseb J. 2008;22:2416–2426. 18326786 10.1096/fj.07-102723

[15] Videnska P, Faldynova M, Juricova H, Babak V, Sisak F, Havlickova H, Rychlik I. Chicken faecal microbiota and disturbances induced by single or repeated therapy with tetracycline and streptomycin. BMC Vet Res. 2013;9:30. 23406343 10.1186/1746-6148-9-30 PMC3598895

[16] M-a P, Vasquez N, Balamurugan R, Pereira E, Dossou-Yovo F, Suau A, Pochart P, Magne F. Metronidazole effects on microbiota and mucus layer thickness in the rat gut. FEMS Microbiol Ecol. 2010;73:601–610. 20579100 10.1111/j.1574-6941.2010.00916.x

[17.] Russell SL, Gold MJ, Hartmann M, Willing BP, Thorson L, Wlodarska M, et al. Early life antibiotic‐driven changes in microbiota enhance susceptibility to allergic asthma. EMBO Rep 2012;13:440–447. 22422004 10.1038/embor.2012.32 PMC3343350

[18] Theriot CM, Koenigsknecht MJ, Carlson JPE, Hatton GE, Nelson AM, Li B, Huffnagle GB, Li JZ, Young VB. Antibiotic-induced shifts in the mouse gut microbiome and metabolome increase susceptibility to Clostridium difficile infection. Nat Commun. 2014;5:3114. 24445449 10.1038/ncomms4114 PMC3950275

[19] Ferreyra JA, Wu KJ, Hryckowian AJ, Bouley DM, Weimer BC, Sonnenburg JL. Gut microbiota-produced succinate promotes C. difficile infection after antibiotic treatment or motility disturbance. Cell Host Microbe. 2014;16:770–777. 25498344 10.1016/j.chom.2014.11.003 PMC4859344

[20] Buffie CG, Jarchum I, Equinda M, Lipuma L, Gobourne A, Viale A, Ubeda C, Xavier J, Pamer EG. Profound alterations of intestinal microbiota following a single dose of clindamycin results in sustained susceptibility to Clostridium difficile-induced colitis. Infect Immun. 2012;80:62–73. 22006564 10.1128/IAI.05496-11 PMC3255689

[21] Reese AT, Cho EH, Klitzman B, Nichols SP, Wisniewski NA, Villa MM, Durand HK, Jiang S, Midani FS, Nimmagadda SN, et al. Antibiotic-induced changes in the microbiota disrupt redox dynamics in the gut. Elife. 2018;7:e35987. 29916366 10.7554/eLife.35987 PMC6008055

[22] Ponziani FR, Scaldaferri F, Petito V, Sterbini FP, Pecere S, Lopetuso LR, Palladini A, Gerardi V, Masucci L, Pompili M, et al. The role of antibiotics in gut microbiota modulation: the eubiotic effects of rifaximin. Dig Dis. 2016;34:269–278. 27027301 10.1159/000443361

[23] Hwang I, Park YJ, Kim Y-R, Kim YN, Ka S, Lee HY, Seong JK, Seok YJ, Kim JB. Alteration of gut microbiota by vancomycin and bacitracin improves insulin resistance via glucagon-like peptide 1 in diet-induced obesity. Faseb J. 2015;29:2397–2411. 25713030 10.1096/fj.14-265983

[24] Fernandez-Luqueno F, López-Valdez F, Gamero-Melo P, Luna-Suárez S, Aguilera-González E, Martínez A, García-Guillermo MD, Hernández-Martínez G, Herrera-Mendoza R, Álvarez-Garza MA, et al. Heavy metal pollution in drinking water-a global risk for human health: a review. African Journal of Environmental Science and Technology. 2013;7:567–584.

[25] Rubin SSD, Alava P, Zekker I, Du Laing G. Van de Wiele T. Arsenic thiolation and the role of sulfate-reducing bacteria from the human intestinal tract. Environ Health Perspect. 2014;122:817. 24833621 10.1289/ehp.1307759 PMC4123032

[26] Van De Wiele T, Gallawa CM, Kubachk KM, Creed JT, Basta N, Dayton EA, Whitacre S, Laing GD, Bradham K. Arsenic metabolism by human gut microbiota upon in vitro digestion of contaminated soils. Environ Health Perspect. 2010;118:1004. 20603239 10.1289/ehp.0901794 PMC2920899

[27] Chi L, Xue J, Tu P, Lai Y, Ru H, Lu K. Gut microbiome disruption altered the biotransformation and liver toxicity of arsenic in mice. Arch Toxicol. 2018;93:25-35–. 10.1007/s00204-018-2332-7 PMC7727877 30357543

[28] Lu K, Ryan PA, Schlieper KA, Graffam ME, Levine S, Wishnok JS, Swenberg JA, Tannenbaum SR, Fox JG. Arsenic exposure perturbs the gut microbiome and its metabolic profile in mice: an integrated metagenomics and metabolomics analysis. Environ Health Perspect. 2014;122:284–291. 24413286 10.1289/ehp.1307429 PMC3948040

[29] Chi L, Bian X, Gao B, Tu P, Ru H, Lu K. The Effects of an Environmentally Relevant Level of Arsenic on the Gut Microbiome and Its Functional Metagenome. Toxicol Sci. 2017;160:193–204. 28973555 10.1093/toxsci/kfx174 PMC5837326

[30] Dheer R, Patterson J, Dudash M, Stachler EN, Bibby KJ, Stolz DB, Shiva S, Wang Z, Hazen SL, Barchowsky A, et al. Arsenic induces structural and compositional colonic microbiome change and promotes host nitrogen and amino acid metabolism. Toxicol Appl Pharmacol. 2015;289:397–408. 26529668 10.1016/j.taap.2015.10.020 PMC4662606

[31] Chi L, Bian X, Gao B, Ru H, Tu P, Lu K. Sex-specific Effects of Arsenic Exposure on the Trajectory and Function of the Gut Microbiome. Chem Res Toxicol. 2016;29:949–951. 27268458 10.1021/acs.chemrestox.6b00066 PMC5079644

[32] Hoen AG, Madan JC, Li Z, Coker M, Lundgren SN, Morrison HG, Palys T, Jackson BP, Sogin ML, Cottingham KL, et al. Sex-specific associations of infants’ gut microbiome with arsenic exposure in a US population. Sci Rep. 2018;8:12627. 30135504 10.1038/s41598-018-30581-9 PMC6105615

[33] Xia J, Lu L, Jin C, Wang S, Zhou J, Ni Y, Fu Z, Jin Y. Effects of short term lead exposure on gut microbiota and hepatic metabolism in adult zebrafish. Comparative Biochemistry and Physiology Part C: Toxicology & Pharmacology. 2018;209:1–8. 29574035 10.1016/j.cbpc.2018.03.007

[34] Xia J, Jin C, Pan Z, Sun L, Fu Z, Jin Y. Chronic exposure to low concentrations of lead induces metabolic disorder and dysbiosis of the gut microbiota in mice. Sci Total Environ. 2018;631:439–448. 29529432 10.1016/j.scitotenv.2018.03.053

[35] Gao B, Chi L, Mahbub R, Bian X, Tu P, Ru H, Lu K. Multi-Omics Reveals that Lead Exposure Disturbs Gut Microbiome Development, Key Metabolites, and Metabolic Pathways. Chem Res Toxicol. 2017;30:996–1005. 28234468 10.1021/acs.chemrestox.6b00401 PMC5654721

[36] Wu J, Wen XW, Faulk C, Boehnke K, Zhang H, Dolinoy DC, Xi C. Perinatal lead exposure alters gut microbiota composition and results in sex-specific bodyweight increases in adult mice. Toxicol Sci. 2016;151:324–333. 26962054 10.1093/toxsci/kfw046 PMC4880136

[37] Breton J, Massart S, Vandamme P, De Brandt E, Pot B, Foligné B. Ecotoxicology inside the gut: impact of heavy metals on the mouse microbiome. BMC Pharmacol Toxicol. 2013;14:62. 24325943 10.1186/2050-6511-14-62 PMC3874687

[38] Liu Y, Li Y, Liu K, Shen J. Exposing to cadmium stress cause profound toxic effect on microbiota of the mice intestinal tract. PLoS ONE. 2014;9:e85323. 24498261 10.1371/journal.pone.0085323 PMC3911910

[39] Zhang S, Jin Y, Zeng Z, Liu Z, Fu Z. Subchronic exposure of mice to cadmium perturbs their hepatic energy metabolism and gut microbiome. Chem Res Toxicol. 2015;28:2000–2009. 26352046 10.1021/acs.chemrestox.5b00237

[40] Ba Q, Li M, Chen P, Huang C, Duan X, Lu L, Li J, Chu R, Xie D, Song H, et al. Sex-dependent effects of cadmium exposure in early life on gut microbiota and fat accumulation in mice. Environ Health Perspect. 2016;125:437–446. 27634282 10.1289/EHP360 PMC5332190

[41] Motta EV, Raymann K, Moran NA. Glyphosate perturbs the gut microbiota of honey bees. Proc Natl Acad Sci USA. 2018;115:10305–10310. 30249635 10.1073/pnas.1803880115 PMC6187125

[42] Aitbali Y, Ba-m’hamed S, Elhidar N, Nafis A, Soraa N, Bennis M. Glyphosate based-herbicide exposure affects gut microbiota, anxiety and depression-like behaviors in mice. Neurotoxicol Teratol. 2018;67:44–49. 29635013 10.1016/j.ntt.2018.04.002

[43] Samsel A, Seneff S. Glyphosate’s suppression of cytochrome P450 enzymes and amino acid biosynthesis by the gut microbiome: pathways to modern diseases. Entropy. 2013;15:1416–1463.

[44] Gao B, Bian X, Mahbub R, Lu K. Sex-specific effects of organophosphate diazinon on the gut microbiome and its metabolic functions. Environ Health Perspect. 2017;125:198–206. 27203275 10.1289/EHP202 PMC5289904

[45] Crisol-Martínez E, Moreno-Moyano LT, Wilkinson N, Prasai T, Brown PH, Moore RJ, Stanley D. A low dose of an organophosphate insecticide causes dysbiosis and sex-dependent responses in the intestinal microbiota of the Japanese quail (Coturnix japonica). PeerJ. 2016;4:e2002. 27168998 10.7717/peerj.2002 PMC4860294

[46] Velmurugan G, Ramprasath T, Swaminathan K, Mithieux G, Rajendhran J, Dhivakar M, Parthasarathy A, Babu DV, Thumburaj LJ, Freddy AJ, et al. Gut microbial degradation of organophosphate insecticides-induces glucose intolerance via gluconeogenesis. Genome Biol. 2017;18:8. 28115022 10.1186/s13059-016-1134-6 PMC5260025

[47] Tu P, Gao B, Chi L, Lai Y, Bian X, Ru H, Lu K. Subchronic low-dose 2, 4-D exposure changed plasma acylcarnitine levels and induced gut microbiome perturbations in mice. Sci Rep. 2019;9:4363. 30867497 10.1038/s41598-019-40776-3 PMC6416245

[48] Zhao Y, Zhang Y, Wang G, Han R, Xie X. Effects of chlorpyrifos on the gut microbiome and urine metabolome in mouse (Mus musculus). Chemosphere. 2016;153:287–293. 27018521 10.1016/j.chemosphere.2016.03.055

[49] Liang Y, Zhan J, Liu D, Luo M, Han J, Liu X, Liu C, Cheng Z, Zhou Z, Wang P. Organophosphorus pesticide chlorpyrifos intake promotes obesity and insulin resistance through impacting gut and gut microbiota. Microbiome. 2019;7:19. 30744700 10.1186/s40168-019-0635-4 PMC6371608

[50] Jin C, Zeng Z, Fu Z, Jin Y. Oral imazalil exposure induces gut microbiota dysbiosis and colonic inflammation in mice. Chemosphere. 2016;160:349–358. 27393971 10.1016/j.chemosphere.2016.06.105

[51] Jin C, Luo T, Zhu Z, Pan Z, Yang J, Wang W, Fu Z, Jin Y. Imazalil exposure induces gut microbiota dysbiosis and hepatic metabolism disorder in zebrafish. Comparative Biochemistry and Physiology Part C: Toxicology & Pharmacology. 2017;202:85–93. 28888875 10.1016/j.cbpc.2017.08.007

[52] Jin C, Xia J, Wu S, Tu W, Pan Z, Fu Z, Wang Y, Jin Y. Insights into a possible influence on gut microbiota and intestinal barrier function during chronic exposure of mice to imazalil. Toxicol Sci. 2017;162:113–123. 10.1093/toxsci/kfx227 29106682

[53] Yuan X, Pan Z, Jin C, Ni Y, Fu Z, Jin Y. Gut microbiota: an underestimated and unintended recipient for pesticide-induced toxicity. Chemosphere. 2019:227:425-434. 10.1016/j.chemosphere.2019.04.088 31003127

[54] Chattopadhyay S, Raychaudhuri U, Chakraborty R. Artificial sweeteners–a review. J Food Sci Technol. 2014;51:611–621. 24741154 10.1007/s13197-011-0571-1 PMC3982014

[55] Bian X, Tu P, Chi L, Gao B, Ru H, Lu K. Saccharin induced liver inflammation in mice by altering the gut microbiota and its metabolic functions. Food Chem Toxicol. 2017:107:530-539. 10.1016/j.fct.2017.04.045 PMC5647777 28472674

[56] Daly K, Darby AC, Hall N, Nau A, Bravo D, Shirazi-Beechey SP. Dietary supplementation with lactose or artificial sweetener enhances swine gut Lactobacillus population abundance. Br J Nutr. 2014;111:S30–S5. 24382146 10.1017/S0007114513002274

[57] Daly K, Darby AC, Hall N, Wilkinson MC, Pongchaikul P, Bravo D, Shirazi‐Beechey SP. Bacterial sensing underlies artificial sweetener‐induced growth of gut L actobacillus. Environ Microbiol. 2016;18:2159–2171. 26058469 10.1111/1462-2920.12942

[58] Bian X, Chi L, Gao B, Tu P, Ru H, Lu K. Gut microbiome response to sucralose and its potential role in inducing liver inflammation in mice. Front Physiol. 2017;8:487. 28790923 10.3389/fphys.2017.00487 PMC5522834

[59] Uebanso T, Ohnishi A, Kitayama R, Yoshimoto A, Nakahashi M, Shimohata T, Mawatari K, Takahashi A. Effects of low-dose non-caloric sweetener consumption on gut microbiota in mice. Nutrients. 2017;9:560. 10.3390/nu9060560 PMC5490539 28587159

[60] Palmnäs MS, Cowan TE, Bomhof MR, Su J, Reimer RA, Vogel HJ, Hittel DS, Shearer J. Low-dose aspartame consumption differentially affects gut microbiota-host metabolic interactions in the diet-induced obese rat. PLoS ONE. 2014;9:e109841. 25313461 10.1371/journal.pone.0109841 PMC4197030

[61] Bian X, Chi L, Gao B, Tu P, Ru H, Lu K. The artificial sweetener acesulfame potassium affects the gut microbiome and body weight gain in CD-1 mice. PLoS ONE. 2017;12:e0178426. 28594855 10.1371/journal.pone.0178426 PMC5464538

[62] Frankenfeld CL, Sikaroodi M, Lamb E, Shoemaker S, Gillevet PM. High-intensity sweetener consumption and gut microbiome content and predicted gene function in a cross-sectional study of adults in the United States. Ann Epidemiol. 2015;25:e4. 10.1016/j.annepidem.2015.06.083 26272781

[63] Lobach AR, Roberts A, Rowland IR. Assessing the in vivo data on low/no-calorie sweeteners and the gut microbiota. Food Chem Toxicol. 2018:124:385-399. 10.1016/j.fct.2018.12.005 30557670

[64] Ruiz-Ojeda FJ, Plaza-Díaz J, Sáez-Lara MJ, Gil A. Effects of sweeteners on the gut microbiota: a review of experimental studies and clinical trials. Advances in Nutrition. 2019;10:S31–S48. 30721958 10.1093/advances/nmy037 PMC6363527

[65] Le Bastard Q, Al‐Ghalith G, Grégoire M, Chapelet G, Javaudin F, Dailly E, Batard E, Knights D, Montassier E. Systematic review: human gut dysbiosis induced by non‐antibiotic prescription medications. Aliment Pharmacol Ther. 2018;47:332–345. 29205415 10.1111/apt.14451

[66] Maier L, Pruteanu M, Kuhn M, Zeller G, Telzerow A, Anderson EE, Brochado AR, Fernandez KC, Dose H, Mori H, et al. Extensive impact of non-antibiotic drugs on human gut bacteria. Nature. 2018;555:623. 29555994 10.1038/nature25979 PMC6108420

[67] Choi JJ, Eum SY, Rampersaud E, Daunert S, Abreu MT, Toborek M. Exercise attenuates PCB-induced changes in the mouse gut microbiome. Environmental Health Perspectives (Online). 2013;121:725. 10.1289/ehp.1306534 PMC3672930 23632211

[68] Zhang L, Nichols RG, Correll J, Murray IA, Tanaka N, Smith PB, Hubbard TD, Sebastian A, Albert I, Hatzakis E, et al. Persistent organic pollutants modify gut microbiota–host metabolic homeostasis in mice through aryl hydrocarbon receptor activation. Environ Health Perspect. 2015;123:679–688. 25768209 10.1289/ehp.1409055 PMC4492271

[69] Lefever DE, Xu J, Chen Y, Huang G, Tamas N, Guo TL. TCDD modulation of gut microbiome correlated with liver and immune toxicity in streptozotocin (STZ)-induced hyperglycemic mice. Toxicol Appl Pharmacol. 2016;304:48–58. 27221631 10.1016/j.taap.2016.05.016 PMC5694619

[70] Gaulke CA, Barton CL, Proffitt S, Tanguay RL, Sharpton TJ. Triclosan exposure is associated with rapid restructuring of the microbiome in adult zebrafish. PLoS ONE. 2016;11:e0154632. 27191725 10.1371/journal.pone.0154632 PMC4871530

[71] Narrowe AB, Albuthi-Lantz M, Smith EP, Bower KJ, Roane TM, Vajda AM, Miller CS. Perturbation and restoration of the fathead minnow gut microbiome after low-level triclosan exposure. Microbiome. 2015;3:6. 25815185 10.1186/s40168-015-0069-6 PMC4374533

[72] Gao B, Tu P, Bian X, Chi L, Ru H, Lu K. Profound perturbation induced by triclosan exposure in mouse gut microbiome: a less resilient microbial community with elevated antibiotic and metal resistomes. BMC Pharmacol Toxicol. 2017;18:46. 28606169 10.1186/s40360-017-0150-9 PMC5469155

[73] Chi L, Mahbub R, Gao B, Bian X, Tu P, Ru H, Lu K. Nicotine Alters the Gut Microbiome and Metabolites of Gut–Brain Interactions in a Sex-Specific Manner. Chem Res Toxicol. 2017;30:2110–2119. 29035044 10.1021/acs.chemrestox.7b00162

[74] Greenblum S, Carr R, Borenstein E. Extensive strain-level copy-number variation across human gut microbiome species. Cell. 2015;160:583–594. 25640238 10.1016/j.cell.2014.12.038 PMC4507803

[75] Wu S, Rhee K-J, Albesiano E, Rabizadeh S, Wu X, Yen H-R, Huso DL, Brancati FL, Wick E, McAllister F, et al. A human colonic commensal promotes colon tumorigenesis via activation of T helper type 17 T cell responses. Nat Med. 2009;15:1016. 19701202 10.1038/nm.2015 PMC3034219

[76] Sekirov I, Russell SL, Antunes LCM, Finlay BB. Gut microbiota in health and disease. Physiol Rev. 2010;90:859–904. 20664075 10.1152/physrev.00045.2009

[77] Lozupone CA, Stombaugh JI, Gordon JI, Jansson JK, Knight R. Diversity, stability and resilience of the human gut microbiota. Nature. 2012;489:220. 22972295 10.1038/nature11550 PMC3577372

[78] Vital M, Howe AC, Tiedje JM. Revealing the bacterial butyrate synthesis pathways by analyzing (meta) genomic data. MBio. 2014;5:e00889–14. 24757212 10.1128/mBio.00889-14 PMC3994512

[79] Vital M, Karch A, Pieper DH. Colonic butyrate-producing communities in humans: an overview using omics data. Msystems. 2017;2:e00130–17. 29238752 10.1128/mSystems.00130-17 PMC5715108

[80] Laukens D, Brinkman BM, Raes J, De Vos M, Vandenabeele P. Heterogeneity of the gut microbiome in mice: guidelines for optimizing experimental design. FEMS Microbiol Rev. 2015;40:117–132. 26323480 10.1093/femsre/fuv036 PMC4703068

[81] Franklin CL, Ericsson AC. Microbiota and reproducibility of rodent models. Lab Anim (NY). 2017;46:114. 28328896 10.1038/laban.1222 PMC5762113

[82] Ericsson AC, Gagliardi J, Bouhan D, Spollen WG, Givan SA, Franklin CL. The influence of caging, bedding, and diet on the composition of the microbiota in different regions of the mouse gut. Sci Rep. 2018;8:4065. 29511208 10.1038/s41598-018-21986-7 PMC5840362

[83] Hildebrand F, Nguyen TLA, Brinkman B, Yunta RG, Cauwe B, Vandenabeele P, Liston A, Raes J.. Inflammation-associated enterotypes, host genotype, cage and inter-individual effects drive gut microbiota variation in common laboratory mice. Genome Biol. 2013;14:R4. 23347395 10.1186/gb-2013-14-1-r4 PMC4053703

[84] Zhang X, Li L, Butcher J, Stintzi A, Figeys D. Advancing functional and translational microbiome research using meta-omics approaches. Microbiome. 2019;7:154. 31810497 10.1186/s40168-019-0767-6 PMC6898977

[85] Wang Q, Wang K, Wu W, Giannoulatou E, Ho JW, Li L. Host and microbiome multi-omics integration: applications and methodologies. Biophys Rev. 2019;11:55–65. 30627872 10.1007/s12551-018-0491-7 PMC6381360

[86] Pearce SC, Coia HG, Karl JP, Pantoja-Feliciano IG, Zachos NC, Intestinal RK. In Vitro and Ex Vivo Models to Study Host-Microbiome Interactions. Front Physiol. 2018;9:1584. 30483150 10.3389/fphys.2018.01584 PMC6240795

[87] Subramanian S, Huq S, Yatsunenko T, Haque R, Mahfuz M, Alam MA, Benezra A, DeStefano J, Meier MF, Muegge BD, et al. Persistent gut microbiota immaturity in malnourished Bangladeshi children. Nature. 2014;510:417. 24896187 10.1038/nature13421 PMC4189846

[88] Blanton LV, Charbonneau MR, Salih T, Barratt MJ, Venkatesh S, Ilkaveya O, Subramanian S, Manary MJ, Trehan I, Jorgensen JM, et al. Gut bacteria that prevent growth impairments transmitted by microbiota from malnourished children. Science. 2016;351:aad3311. 26912898 10.1126/science.aad3311 PMC4787260

[89] Raman AS, Gehrig JL, Venkatesh S, Chang H-W, Hibberd MC, Subramanian S, Kang G, Bessong PO, Lima AA, Kosek MN, et al. A sparse covarying unit that describes healthy and impaired human gut microbiota development. Science. 2019;365:eaau4735. 31296739 10.1126/science.aau4735 PMC6683326

[90] Gehrig JL, Venkatesh S, Chang H-W, Hibberd MC, Kung VL, Cheng J, Chen RY, Subramanian S, Cowardin CA, Meier MF, et al. Effects of microbiota-directed foods in gnotobiotic animals and undernourished children. Science. 2019;365:eaau4732. 31296738 10.1126/science.aau4732 PMC6683325

[91] Nicolas GR, Chang PV. Deciphering the Chemical Lexicon of Host–Gut Microbiota Interactions. Trends Pharmacol Sci. 2019;40:430-445.. 10.1016/j.tips.2019.04.006 PMC6681900 31079848

[92] Bergman E. Energy contributions of volatile fatty acids from the gastrointestinal tract in various species. Physiol Rev. 1990;70:567–590. 2181501 10.1152/physrev.1990.70.2.567

[93] Wiltrout D, Satter L. Contribution of propionate to glucose synthesis in the lactating and nonlactating cow. J Dairy Sci. 1972;55:307–317. 5062250 10.3168/jds.S0022-0302(72)85487-0

[94] Den Besten G, Van Eunen K, Groen AK, Venema K, Reijngoud D-J, Bakker BM. The role of short-chain fatty acids in the interplay between diet, gut microbiota, and host energy metabolism. J Lipid Res. 2013;54:2325–2340. 23821742 10.1194/jlr.R036012 PMC3735932

[95] Schwiertz A, Taras D, Schäfer K, Beijer S, Bos NA, Donus C, Hardt PD. Microbiota and SCFA in lean and overweight healthy subjects. Obesity. 2010;18:190–195. 19498350 10.1038/oby.2009.167

[96] Turnbaugh PJ, Ley RE, Mahowald MA, Magrini V, Mardis ER, Gordon JI. An obesity-associated gut microbiome with increased capacity for energy harvest. Nature. 2006;444:1027–1131. 17183312 10.1038/nature05414

[97] Ratajczak W, Rył A, Mizerski A, Walczakiewicz K, Sipak O, Laszczyńska M. Immunomodulatory potential of gut microbiome-derived short-chain fatty acids (SCFAs). Acta Biochim Pol. 2019;66:1–12. 30831575 10.18388/abp.2018_2648

[98] Le Poul E, Loison C, Struyf S, Springael J-Y, Lannoy V, Decobecq M-E, Brezillon S, Dupriez V, Vassart G, Van Damme J, et al. Functional characterization of human receptors for short chain fatty acids and their role in polymorphonuclear cell activation. J Biol Chem. 2003;278:25481–25489. 12711604 10.1074/jbc.M301403200

[99] Smith PM, Howitt MR, Panikov N, Michaud M, Gallini CA, Bohlooly-Y M, Glickman JN, Garrett WS. The microbial metabolites, short-chain fatty acids, regulate colonic Treg cell homeostasis. Science. 2013;341(6145):569–573. doi:10.1126/science.1241165. 23828891 PMC3807819

[100] Mariño E, Richards JL, McLeod KH, Stanley D, Yap YA, Knight J, McKenzie C, Kranich J, Oliveira AC, Rossello FJ, et al. Gut microbial metabolites limit the frequency of autoimmune T cells and protect against type 1 diabetes. Nat Immunol. 2017;18:552. 28346408 10.1038/ni.3713

[101] Okada T, Fukuda S, Hase K, Nishiumi S, Izumi Y, Yoshida M, Hagiwara T, Kawashima R, Yamazaki M, Oshio T, et al. Microbiota-derived lactate accelerates colon epithelial cell turnover in starvation-refed mice. Nat Commun. 2013;4:1654. 23552069 10.1038/ncomms2668

[102] Kimura I, Ozawa K, Inoue D, Imamura T, Kimura K, Maeda T, Terasawa K, Kashihara D, Hirano K, Tani T, et al. The gut microbiota suppresses insulin-mediated fat accumulation via the short-chain fatty acid receptor GPR43. Nat Commun. 2013;4:1829. 23652017 10.1038/ncomms2852 PMC3674247

[103] Nakajima A, Nakatani A, Hasegawa S, Irie J, Ozawa K, Tsujimoto G, Suganami T, Itoh H, Kimura I. The short chain fatty acid receptor GPR43 regulates inflammatory signals in adipose tissue M2-type macrophages. PLoS ONE. 2017;12:e0179696. 28692672 10.1371/journal.pone.0179696 PMC5503175

[104] Trompette A, Gollwitzer ES, Yadava K, Sichelstiel AK, Sprenger N, Ngom-Bru C, Blanchard C, Junt T, Nicod LP, Harris NL, et al. Gut microbiota metabolism of dietary fiber influences allergic airway disease and hematopoiesis. Nat Med. 2014;20:159. 24390308 10.1038/nm.3444

[105] Kobayashi M, Mikami D, Kimura H, Kamiyama K, Morikawa Y, Yokoi S, Kasuno K, Takahashi N, Taniguchi T, Iwano M. Short-chain fatty acids, GPR41 and GPR43 ligands, inhibit TNF-α-induced MCP-1 expression by modulating p38 and JNK signaling pathways in human renal cortical epithelial cells. Biochem Biophys Res Commun. 2017;486:499–505. 28322790 10.1016/j.bbrc.2017.03.071

[106] Singh N, Gurav A, Sivaprakasam S, Brady E, Padia R, Shi H, Thangaraju M, Prasad PD, Manicassamy S, Munn DH, et al. Activation of Gpr109a, receptor for niacin and the commensal metabolite butyrate, suppresses colonic inflammation and carcinogenesis. Immunity. 2014;40:128–139. 24412617 10.1016/j.immuni.2013.12.007 PMC4305274

[107] Docampo MD, Stein-Thoeringer CK, Lazrak A, Mdb DS, Cross J. van den Brink MR. Expression of the Butyrate/Niacin Receptor, GPR109a on T Cells Plays an Important Role in a Mouse Model of Graft Versus Host Disease. Am Soc Hematology. 2018;132:61.

[108] Tan J, McKenzie C, Vuillermin PJ, Goverse G, Vinuesa CG, Mebius RE, Macia L, Mackay CR.. Dietary fiber and bacterial SCFA enhance oral tolerance and protect against food allergy through diverse cellular pathways. Cell Rep. 2016;15:2809–2824. 27332875 10.1016/j.celrep.2016.05.047

[109] Arpaia N, Campbell C, Fan X, Dikiy S, Van Der Veeken J, Deroos P, Liu H, Cross JR, Pfeffer K, Coffer PJ, et al. Metabolites produced by commensal bacteria promote peripheral regulatory T-cell generation. Nature. 2013;504:451. 24226773 10.1038/nature12726 PMC3869884

[110] Chang PV, Hao L, Offermanns S, Medzhitov R. The microbial metabolite butyrate regulates intestinal macrophage function via histone deacetylase inhibition. Proc Natl Acad Sci USA. 2014;111:2247–2252. 24390544 10.1073/pnas.1322269111 PMC3926023

[111] Schulthess J, Pandey S, Capitani M, Rue-Albrecht KC, Arnold I, Franchini F, Chomka A, Ilott NE, Johnston DG, Pires E, et al. The short chain fatty acid butyrate imprints an antimicrobial program in macrophages. Immunity. 2019;50(432–45):e7. 10.1016/j.immuni.2018.12.018 PMC6382411 30683619

[112] Mathewson ND, Jenq R, Mathew AV, Koenigsknecht M, Hanash A, Toubai T, Oravecz-Wilson K, Wu SR, Sun Y, Rossi C, et al. Gut microbiome–derived metabolites modulate intestinal epithelial cell damage and mitigate graft-versus-host disease. Nat Immunol. 2016;17:505. 26998764 10.1038/ni.3400 PMC4836986

[113] Byndloss MX, Olsan EE, Rivera-Chávez F, Tiffany CR, Cevallos SA, Lokken KL, Torres TP, Byndloss AJ, Faber F, Gao Y, et al. Microbiota-activated PPAR-γ signaling inhibits dysbiotic Enterobacteriaceae expansion. Science. 2017;357:570–575. 28798125 10.1126/science.aam9949 PMC5642957

[114] Rivera-Chávez F, Zhang LF, Faber F, Lopez CA, Byndloss MX, Olsan EE, Xu G, Velazquez EM, Lebrilla CB, Winter SE, et al. Depletion of butyrate-producing Clostridia from the gut microbiota drives an aerobic luminal expansion of Salmonella. Cell Host Microbe. 2016;19:443–454. 27078066 10.1016/j.chom.2016.03.004 PMC4832419

[115] Den Besten G, Bleeker A, Gerding A, Van Eunen K, Havinga R, Van Dijk TH, Oosterveer MH, Jonker JW, Groen AK, Reijngoud DJ, et al. Short-chain fatty acids protect against high-fat diet–induced obesity via a PPARγ-dependent switch from lipogenesis to fat oxidation. Diabetes. 2015;64:2398–2408. 25695945 10.2337/db14-1213

[116] Hung CC, Garner CD, Slauch JM, Dwyer ZW, Lawhon SD, Frye JG, McClelland M, Ahmer BM, Altier C. The intestinal fatty acid propionate inhibits S almonella invasion through the post‐translational control of HilD. Mol Microbiol. 2013;87:1045–1060. 23289537 10.1111/mmi.12149 PMC3581741

[117] Ridlon JM, Kang D-J, Hylemon PB. Bile salt biotransformations by human intestinal bacteria. J Lipid Res. 2006;47:241–259. 16299351 10.1194/jlr.R500013-JLR200

[118] Swann JR, Want EJ, Geier FM, Spagou K, Wilson ID, Sidaway JE, Nicholson JK, Holmes E. Systemic gut microbial modulation of bile acid metabolism in host tissue compartments. Proc Natl Acad Sci USA. 2011;108:4523–4530. 20837534 10.1073/pnas.1006734107 PMC3063584

[119] Lee FY, Lee H, Hubbert ML, Edwards PA, Zhang Y. FXR, a multipurpose nuclear receptor. Trends Biochem Sci. 2006;31:572–580. 16908160 10.1016/j.tibs.2006.08.002

[120] Wang H, Chen J, Hollister K, Sowers LC, Forman BM. Endogenous bile acids are ligands for the nuclear receptor FXR/BAR. Mol Cell. 1999;3:543–553. 10360171 10.1016/s1097-2765(00)80348-2

[121] Sayin SI, Wahlström A, Felin J, Jäntti S, Marschall H-U, Bamberg K,Angelin B, Hyötyläinen T, Orešič M, Bäckhed F. Gut microbiota regulates bile acid metabolism by reducing the levels of tauro-beta-muricholic acid, a naturally occurring FXR antagonist. Cell Metab. 2013;17:225–235. 23395169 10.1016/j.cmet.2013.01.003

[122] Li F, Jiang C, Krausz KW, Li Y, Albert I, Hao H, Fabre KM, Mitchell JB, Patterson AD, Gonzalez FJ. Microbiome remodelling leads to inhibition of intestinal farnesoid X receptor signalling and decreased obesity. Nat Commun. 2013;4:2384. 24064762 10.1038/ncomms3384 PMC6595219

[123] Tu H, Okamoto AY, Shan B. FXR, a bile acid receptor and biological sensor. Trends Cardiovasc Med. 2000;10:30–35. 11150726 10.1016/s1050-1738(00)00043-8

[124] Jiang C, Xie C, Li F, Zhang L, Nichols RG, Krausz KW, Cai J, Qi Y, Fang ZZ, Takahashi S, et al. Intestinal farnesoid X receptor signaling promotes nonalcoholic fatty liver disease. The Journal of Clinical Investigation. 2015;125:386–402. 25500885 10.1172/JCI76738 PMC4382255

[125] Parséus A, Sommer N, Sommer F, Caesar R, Molinaro A, Ståhlman M, Greiner TU, Perkins R, Bäckhed F. Microbiota-induced obesity requires farnesoid X receptor. Gut. 2017;66:429–437. 26740296 10.1136/gutjnl-2015-310283 PMC5534765

[126] Jain U, Lai C-W, Xiong S, Goodwin VM, Lu Q, Muegge BD, Christophi GP, VanDussen KL, Cummings BP, Young E, et al. Temporal regulation of the bacterial metabolite deoxycholate during colonic repair is critical for crypt regeneration. Cell Host Microbe. 2018;24(353–63):e5. 10.1016/j.chom.2018.07.019 PMC6555552 30122655

[127] Sato H, Genet C, Strehle A, Thomas C, Lobstein A, Wagner A, Mioskowski C, Auwerx J, Saladin R. Anti-hyperglycemic activity of a TGR5 agonist isolated from Olea europaea. Biochem Biophys Res Commun. 2007;362:793–798. 17825251 10.1016/j.bbrc.2007.06.130

[128] Yanguas‐Casás N, Barreda‐Manso MA, Nieto‐Sampedro M, Tudca: RL. An agonist of the bile acid receptor GPBAR1/TGR5 with anti‐inflammatory effects in microglial cells. J Cell Physiol. 2017;232:2231–2245. 27987324 10.1002/jcp.25742

[129] Mendes M Neuroprotective effects of TUDCA in Parkinson’s disease: dissecting the anti-oxidant and anti-inflammatory effects of this bile acid in the mouse cerebral cortex. 2017.

[130] Cheng K-C, Chang W-T, Kuo FY, Chen Z-C, Li Y, Cheng J-T. TGR5 activation ameliorates hyperglycemia-induced cardiac hypertrophy in H9c2 cells. Sci Rep. 2019;9:3633. 30842472 10.1038/s41598-019-40002-0 PMC6403401

[131] Yoshimoto S, Loo TM, Atarashi K, Kanda H, Sato S, Oyadomari S, Iwakura Y, Oshima K, Morita H, Hattori M, et al. Obesity-induced gut microbial metabolite promotes liver cancer through senescence secretome. Nature. 2013;499:97. 23803760 10.1038/nature12347

[132] Ma C, Han M, Heinrich B, Fu Q, Zhang Q, Sandhu M, Agdashian D, Terabe M, Berzofsky JA, Fako V, et al. Gut microbiome–mediated bile acid metabolism regulates liver cancer via NKT cells. Science. 2018;360:eaan5931. 29798856 10.1126/science.aan5931 PMC6407885

[133] Staudinger JL, Goodwin B, Jones SA, Hawkins-Brown D, MacKenzie KI, LaTour A, Liu Y, Klaassen CD, Brown KK, Reinhard J, et al. The nuclear receptor PXR is a lithocholic acid sensor that protects against liver toxicity. Proc Natl Acad Sci USA. 2001;98:3369–3374. 11248085 10.1073/pnas.051551698 PMC30660

[134.] Buffie CG, Bucci V, Stein RR, McKenney PT, Ling L, Gobourne A, et al. Precision microbiome reconstitution restores bile acid mediated resistance to Clostridium difficile. Nature 2015;517:205. 25337874 10.1038/nature13828 PMC4354891

[135] Zelante T, Iannitti RG, Cunha C, De Luca A, Giovannini G, Pieraccini G, Zecchi R, D’Angelo C, Massi-Benedetti C, Fallarino F, et al. Tryptophan catabolites from microbiota engage aryl hydrocarbon receptor and balance mucosal reactivity via interleukin-22. Immunity. 2013;39:372–385. 23973224 10.1016/j.immuni.2013.08.003

[136] Agus A, Planchais J, Sokol H. Gut microbiota regulation of tryptophan metabolism in health and disease. Cell Host Microbe. 2018;23:716–724. 29902437 10.1016/j.chom.2018.05.003

[137] Cervantes-Barragan L, Chai JN, Tianero MD, Di Luccia B, Ahern PP, Merriman J, Cortez VS, Caparon MG, Donia MS, Gilfillan S, et al. Lactobacillus reuteri induces gut intraepithelial CD4+ CD8αα+ T cells. Science. 2017;357:806–810. 28775213 10.1126/science.aah5825 PMC5687812

[138.] Krishnan S, Ding Y, Saedi N, Choi M, Sridharan GV, Sherr DH, et al. Gut microbiota-derived tryptophan metabolites modulate inflammatory response in hepatocytes and macrophages. Cell Rep 2018;23:1099–1111. 29694888 10.1016/j.celrep.2018.03.109 PMC6392449

[139] Miani M, Le Naour J, Waeckel-Enée E, Chand Verma S, Straube M, Emond P, Ryffel B, Van Endert P, Sokol H, Diana J. Gut microbiota-stimulated innate lymphoid cells support β-defensin 14 expression in pancreatic endocrine cells, preventing autoimmune diabetes. Cell Metab. 2018;28(557–72):e6. 10.1016/j.cmet.2018.06.012 30017352

[140] Rothhammer V, Mascanfroni ID, Bunse L, Takenaka MC, Kenison JE, Mayo L, Chao CC, Patel B, Yan R, Blain M, et al. Type I interferons and microbial metabolites of tryptophan modulate astrocyte activity and central nervous system inflammation via the aryl hydrocarbon receptor. Nat Med. 2016;22:586. 27158906 10.1038/nm.4106 PMC4899206

[141] Natividad JM, Agus A, Planchais J, Lamas B, Jarry AC, Martin R, Michel ML, Chong-Nguyen C, Roussel R, Straube M, et al. Impaired aryl hydrocarbon receptor ligand production by the gut microbiota is a key factor in metabolic syndrome. Cell Metab. 2018;28(737–49):e4. 10.1016/j.cmet.2018.07.001 30057068

[142] Venkatesh M, Mukherjee S, Wang H, Li H, Sun K, Benechet AP, Qiu Z, Maher L, Redinbo MR, Phillips RS, et al. Symbiotic bacterial metabolites regulate gastrointestinal barrier function via the xenobiotic sensor PXR and Toll-like receptor 4. Immunity. 2014;41:296–310. 25065623 10.1016/j.immuni.2014.06.014 PMC4142105

[143] Chimerel C, Emery E, Summers DK, Keyser U, Gribble FM, Reimann F. Bacterial metabolite indole modulates incretin secretion from intestinal enteroendocrine L cells. Cell Rep. 2014;9:1202–1208. 25456122 10.1016/j.celrep.2014.10.032 PMC4308618

[144] O’Mahony SM, Clarke G, Borre Y, Dinan T, Cryan J. Serotonin, tryptophan metabolism and the brain-gut-microbiome axis. Behav Brain Res. 2015;277:32–48. 25078296 10.1016/j.bbr.2014.07.027

[145] Williams BB, Van Benschoten AH, Cimermancic P, Donia MS, Zimmermann M, Taketani M, Ishihara A, Kashyap PC, Fraser JS, Fischbach MA. Discovery and characterization of gut microbiota decarboxylases that can produce the neurotransmitter tryptamine. Cell Host Microbe. 2014;16:495–503. 25263219 10.1016/j.chom.2014.09.001 PMC4260654

[146] Bhattarai Y, Williams BB, Battaglioli EJ, Whitaker WR, Till L, Grover M, Linden DR, Akiba Y, Kandimalla KK, Zachos NC, et al. Gut microbiota-produced tryptamine activates an epithelial G-protein-coupled receptor to increase colonic secretion. Cell Host Microbe. 2018;23(775–85):e5. 10.1016/j.chom.2018.05.004 PMC6055526 29902441

[147] Yano JM, Yu K, Donaldson GP, Shastri GG, Ann P, Ma L, Nagler CR, Ismagilov RF, Mazmanian SK, Hsiao EY. Indigenous bacteria from the gut microbiota regulate host serotonin biosynthesis. Cell. 2015;161:264–276. 25860609 10.1016/j.cell.2015.02.047 PMC4393509

[148] Martin AM, Yabut JM, Choo JM, Page AJ, Sun EW, Jessup CF, Wesselingh SL, Khan WI, Rogers GB, Steinberg GR, et al. The gut microbiome regulates host glucose homeostasis via peripheral serotonin. Proc Natl Acad Sci USA. 2019;116:19802-19804. 10.1073/pnas.1909311116 PMC6778212 31527237

[149] Tan Y, Kagan JC. A cross-disciplinary perspective on the innate immune responses to bacterial lipopolysaccharide. Mol Cell. 2014;54:212–223. 24766885 10.1016/j.molcel.2014.03.012 PMC4096783

[150] Park BS, Song DH, Kim HM, Choi B-S, Lee H, Lee J-O. The structural basis of lipopolysaccharide recognition by the TLR4–MD-2 complex. Nature. 2009;458:1191. 19252480 10.1038/nature07830

[151] Pasternak BA, D’Mello S, Jurickova II, Han X, Willson T, Flick L, Petiniot L, Uozumi N, Divanovic S, Traurnicht A, et al. Lipopolysaccharide exposure is linked to activation of the acute phase response and growth failure in pediatric Crohn’s disease and murine colitis. Inflamm Bowel Dis. 2009;16:856–869. 10.1002/ibd.21132 PMC3052288 19924809

[152] Cani PD, Amar J, Iglesias MA, Poggi M, Knauf C, Bastelica D, Neyrinck AM, Fava F, Tuohy KM, Chabo C, et al. Metabolic endotoxemia initiates obesity and insulin resistance. Diabetes. 2007;56:1761–1772. 17456850 10.2337/db06-1491

[153] Feehley T, Belda-Ferre P, Nagler CR. What’s LPS got to do with it? A role for gut LPS variants in driving autoimmune and allergic disease. Cell Host Microbe. 2016;19:572–574. 27173923 10.1016/j.chom.2016.04.025

[154] Dasgupta S, Erturk-Hasdemir D, Ochoa-Reparaz J, Reinecker H-C, Kasper DL. Plasmacytoid dendritic cells mediate anti-inflammatory responses to a gut commensal molecule via both innate and adaptive mechanisms. Cell Host Microbe. 2014;15:413–423. 24721570 10.1016/j.chom.2014.03.006 PMC4020153

[155] Johnson JL, Jones MB, Cobb BA. Polysaccharide A from the capsule of Bacteroides fragilis induces clonal CD4+ T cell expansion. J Biol Chem. 2015;290:5007–5014. 25540199 10.1074/jbc.M114.621771 PMC4335237

[156.] Kinjo Y, Wu D, Kim G, Xing G-W, Poles MA, Ho DD, et al. Recognition of bacterial glycosphingolipids by natural killer T cells. Nature 2005;434:520. 15791257 10.1038/nature03407

[157] Mattner J, DeBord KL, Ismail N, Goff RD, Cantu III C, Zhou D, Saint-Mezard P, Wang V, Gao Y, Yin N, et al. Exogenous and endogenous glycolipid antigens activate NKT cells during microbial infections. Nature. 2005;434:525. 15791258 10.1038/nature03408

[158] An D, Oh SF, Olszak T, Neves JF, Avci FY, Erturk-Hasdemir D, Lu X, Zeissig S, Blumberg RS, Kasper DL. Sphingolipids from a symbiotic microbe regulate homeostasis of host intestinal natural killer T cells. Cell. 2014;156:123–133. 24439373 10.1016/j.cell.2013.11.042 PMC3909465

[159] Brown EM, Ke X, Hitchcock D, Jeanfavre S, Avila-Pacheco J, Nakata T, Arthur TD, Fornelos N, Heim C, Franzosa EA, et al. Bacteroides-Derived Sphingolipids Are Critical for Maintaining Intestinal Homeostasis and Symbiosis. Cell Host Microbe. 2019;25(668–80):e7. 10.1016/j.chom.2019.04.002 PMC6544385 31071294

[160] Barcik W, Wawrzyniak M, Akdis CA, O’Mahony L. Immune regulation by histamine and histamine-secreting bacteria. Curr Opin Immunol. 2017;48:108–113. 28923468 10.1016/j.coi.2017.08.011

[161] Thomas CM, Hong T, Van Pijkeren JP, Hemarajata P, Trinh DV, Hu W, Britton RA, Kalkum M, Versalovic J. Histamine derived from probiotic Lactobacillus reuteri suppresses TNF via modulation of PKA and ERK signaling. PLoS ONE. 2012;7:e31951. 22384111 10.1371/journal.pone.0031951 PMC3285189

[162] Gao C, Major A, Rendon D, Lugo M, Jackson V, Shi Z, Mori-Akiyama Y, Versalovic J. Histamine H2 receptor-mediated suppression of intestinal inflammation by probiotic Lactobacillus reuteri. MBio. 2015;6:e01358–15. 26670383 10.1128/mBio.01358-15 PMC4701830

[163] Levy M, Thaiss CA, Zeevi D, Dohnalova L, Zilberman-Schapira G, Mahdi JA, David E, Savidor A, Korem T, Herzig Y, et al. Microbiota-modulated metabolites shape the intestinal microenvironment by regulating NLRP6 inflammasome signaling. Cell. 2015;163:1428–1443. 26638072 10.1016/j.cell.2015.10.048 PMC5665753

[164] Watanabe T, Nishio H, Tanigawa T, Yamagami H, Okazaki H, Watanabe K, Tominaga K, Fujiwara Y, Oshitani N, Asahara T, et al. Probiotic Lactobacillus casei strain Shirota prevents indomethacin-induced small intestinal injury: involvement of lactic acid. American Journal of Physiology-Gastrointestinal and Liver Physiology. 2009;297:G506–G13. 19589943 10.1152/ajpgi.90553.2008

[165] Iraporda C, Errea A, Romanin DE, Cayet D, Pereyra E, Pignataro O, Sirard JC, Garrote GL, Abraham AG, Rumbo M. Lactate and short chain fatty acids produced by microbial fermentation downregulate proinflammatory responses in intestinal epithelial cells and myeloid cells. Immunobiology. 2015;220:1161–1169. 26101138 10.1016/j.imbio.2015.06.004

[166] Offermanns S. Free fatty acid (FFA) and hydroxy carboxylic acid (HCA) receptors. Annu Rev Pharmacol Toxicol. 2014;54:407–434. 24160702 10.1146/annurev-pharmtox-011613-135945

[167] Hoque R, Farooq A, Ghani A, Gorelick F, Mehal WZ. Lactate reduces liver and pancreatic injury in Toll-like receptor–and inflammasome-mediated inflammation via GPR81-mediated suppression of innate immunity. Gastroenterology. 2014;146:1763–1774. 24657625 10.1053/j.gastro.2014.03.014 PMC4104305

[168] Lee Y-S, Kim T-Y, Kim Y, Lee S-H, Kim S, Kang SW, Yang JY, Baek IJ, Sung YH, Park YY, et al. Microbiota-derived lactate accelerates intestinal stem-cell-mediated epithelial development. Cell Host Microbe. 2018;24(833–46):e6. 10.1016/j.chom.2018.11.002 30543778

[169] Morita N, Umemoto E, Fujita S, Hayashi A, Kikuta J, Kimura I, Haneda T, Imai T, Inoue A, Mimuro H, et al. GPR31-dependent dendrite protrusion of intestinal CX3CR1+ cells by bacterial metabolites. Nature. 2019;566:110. 30675063 10.1038/s41586-019-0884-1

[170] Muñoz-Tamayo R, Laroche B, É W, Doré J, Duncan SH, Flint HJ, Leclerc M. Kinetic modelling of lactate utilization and butyrate production by key human colonic bacterial species. FEMS Microbiol Ecol. 2011;76:615–624. 21388423 10.1111/j.1574-6941.2011.01085.x

[171] Wang Z, Klipfell E, Bennett BJ, Koeth R, Levison BS, DuGar B, Feldstein AE, Britt EB, Fu X, Chung YM, et al. Gut flora metabolism of phosphatidylcholine promotes cardiovascular disease. Nature. 2011;472:57. 21475195 10.1038/nature09922 PMC3086762

[172] Koeth RA, Wang Z, Levison BS, Buffa JA, Org E, Sheehy BT, Britt EB, Fu X, Wu Y, Li L, et al. Intestinal microbiota metabolism of L-carnitine, a nutrient in red meat, promotes atherosclerosis. Nat Med. 2013;19:576. 23563705 10.1038/nm.3145 PMC3650111

[173] Zhu W, Gregory JC, Org E, Buffa JA, Gupta N, Wang Z, Li L, Fu X, Wu Y, Mehrabian M, et al. Gut microbial metabolite TMAO enhances platelet hyperreactivity and thrombosis risk. Cell. 2016;165:111–124. 26972052 10.1016/j.cell.2016.02.011 PMC4862743

[174] Dambrova M, Latkovskis G, Kuka J, Strele I, Konrade I, Grinberga S, Hartmane D, Pugovics O, Erglis A, Liepinsh E. Diabetes is associated with higher trimethylamine N-oxide plasma levels. Experimental and Clinical Endocrinology & Diabetes. 2016;124:251–256. 27123785 10.1055/s-0035-1569330

[175] Tang WW, Wang Z, Kennedy DJ, Wu Y, Buffa JA, Agatisa-Boyle B, Li XS, Levison BS, Hazen SL. Gut microbiota-dependent trimethylamine N-oxide (TMAO) pathway contributes to both development of renal insufficiency and mortality risk in chronic kidney disease. Circ Res. 2015;116:448–455. 25599331 10.1161/CIRCRESAHA.116.305360 PMC4312512

[176] Tan X, Liu Y, Long J, Chen S, Liao G, Wu S, Li C, Wang L, Ling W, Zhu H. Trimethylamine N‐Oxide Aggravates Liver Steatosis Through Modulation of Bile Acid Metabolism and Inhibition of Farnesoid X Receptor Signaling in Nonalcoholic Fatty Liver Disease. Molecular nutrition & food research. 2019:63:1900257. 10.1002/mnfr.201900257 31095863

[177] Romano KA, Martinez-del Campo A, Kasahara K, Chittim CL, Vivas EI, Amador-Noguez D, Balskus EP, Rey FE. Metabolic, epigenetic, and transgenerational effects of gut bacterial choline consumption. Cell Host Microbe. 2017;22(279–90):e7. 10.1016/j.chom.2017.07.021 PMC5599363 28844887

[178] Spiga L, Winter MG, De Carvalho TF, Zhu W, Hughes ER, Gillis CC, Behrendt CL, Kim J, Chessa D, Andrews-Polymenis HL, et al. An oxidative central metabolism enables Salmonella to utilize microbiota-derived succinate. Cell Host Microbe. 2017;22(291–301):e6. 10.1016/j.chom.2017.07.018 PMC5599368 28844888

[179] Curtis MM, Hu Z, Klimko C, Narayanan S, Deberardinis R, Sperandio V. The gut commensal Bacteroides thetaiotaomicron exacerbates enteric infection through modification of the metabolic landscape. Cell Host Microbe. 2014;16:759–769. 25498343 10.1016/j.chom.2014.11.005 PMC4269104

[180] Nagao-Kitamoto H, Shreiner AB, Gillilland III MG, Kitamoto S, Ishii C, Hirayama A, Kuffa P, El-Zaatari M, Grasberger H, Seekatz AM, et al. Functional characterization of inflammatory bowel disease–associated gut dysbiosis in gnotobiotic mice. Cellular and Molecular Gastroenterology and Hepatology. 2016;2:468–481. 27795980 10.1016/j.jcmgh.2016.02.003 PMC5042563

[181] Ariake K, Ohkusa T, Sakurazawa T, Kumagai J, Eishi Y, Hoshi S, Yajima T. Roles of mucosal bacteria and succinic acid in colitis caused by dextran sulfate sodium in mice. J Med Dent Sci. 2000;47:233–241. 12160236

[182] Setoyama H, Imaoka A, Ishikawa H, Umesaki Y. Prevention of gut inflammation by Bifidobacterium in dextran sulfate-treated gnotobiotic mice associated with Bacteroides strains isolated from ulcerative colitis patients. Microbes Infect. 2003;5:115–122. 12650769 10.1016/s1286-4579(02)00080-1

[183] Serena C, Ceperuelo-Mallafré V, Keiran N, Queipo-Ortuño MI, Bernal R, Gomez-Huelgas R, Urpi-Sarda M, Sabater M, Pérez-Brocal V, Andrés-Lacueva C, et al. Elevated circulating levels of succinate in human obesity are linked to specific gut microbiota. Isme J. 2018;12:1642. 29434314 10.1038/s41396-018-0068-2 PMC6018807

[184] Perruzza L, Gargari G, Proietti M, Fosso B, D’Erchia AM, Faliti CE, Rezzonico-Jost T, Scribano D, Mauri L, Colombo D, et al. T follicular helper cells promote a beneficial gut ecosystem for host metabolic homeostasis by sensing microbiota-derived extracellular ATP. Cell Rep. 2017;18:2566–2575. 28297661 10.1016/j.celrep.2017.02.061 PMC5368345

[185] Chang Y-L, Rossetti M, Vlamakis H, Casero D, Sunga G, Harre N, Miller S, Humphries R, Stappenbeck T, Simpson KW, et al. A screen of Crohn’s disease-associated microbial metabolites identifies ascorbate as a novel metabolic inhibitor of activated human T cells. Mucosal Immunol. 2019;12:457. 29695840 10.1038/s41385-018-0022-7 PMC6202286

